# Is being in paid work beyond state pension age beneficial for health? Evidence from England using a life-course approach

**DOI:** 10.1136/jech-2016-208086

**Published:** 2017-04-10

**Authors:** Giorgio Di Gessa, Laurie M Corna, Loretta G Platts, Diana Worts, Peggy McDonough, Amanda Sacker, Debora Price, Karen Glaser

**Affiliations:** 1 Department of Social Policy, The London School of Economics and Political Science, London, UK; 2 Institute of Gerontology, Department of Global Health and Social Medicine, King's College London, London, UK; 3 Stress Research Institute, Stockholm University, Stockholm, Sweden; 4 Dalla Lana School of Public Health, University of Toronto, Toronto, Ontario, Canada; 5 Institute of Epidemiology & Health, University College London, London, UK; 6 School of Social Sciences, University of Manchester, Manchester, UK

**Keywords:** EMPLOYMENT, AGEING, OCCUPATIONAL HEALTH, HEALTH STATUS, LONGITUDINAL STUDIES

## Abstract

**Background:**

Given the current policy emphasis in many Western societies on extending working lives, we investigated the health effects of being in paid work beyond state pension age (SPA). Until now, work has largely focused on the health of those who exit the labour force early.

**Methods:**

Our data come from waves 2–4 of the English Longitudinal Study of Ageing, including the life history interview at wave 3. Using logistic and linear regression models, we assessed the longitudinal associations between being in paid work beyond SPA and 3 measures of health (depression, a latent measure of somatic health and sleep disturbance) among men aged 65–74 and women aged 60–69. Our analyses controlled for baseline health and socioeconomic characteristics, as well as for work histories and health in adulthood and childhood.

**Results:**

Approximately a quarter of women and 15% of men were in paid work beyond SPA. Descriptive bivariate analyses suggested that men and women in paid work were more likely to report better health at follow-up. However, once baseline socioeconomic characteristics as well as adulthood and baseline health and labour market histories were accounted for, the health benefits of working beyond SPA were no longer significant.

**Conclusions:**

Potential health benefits of working beyond SPA need to be considered in the light of the fact that those who report good health and are more socioeconomically advantaged are more likely to be working beyond SPA to begin with.

## Introduction

In response to population ageing and the associated rising costs of pensions, health and social care, many Western governments are pursuing policies designed to extend working lives, including raising the state pension age (SPA).^1^ The UK, like several other European countries, increased SPA for women from 60 to 66 by 2020, and for both sexes to 68 by 2046.^2^ Until now, few studies have addressed the health implications of working at later ages, including beyond SPA.

A large body of evidence suggests that paid work in the prime adult years is generally beneficial for physical and psychological health and well-being.^3–5^ However, research on work and health *in later life* has mostly focused on the health effects of the event and/or timing of retirement (ie, age at retirement), and in particular on early retirement broadly referring to exits from the labour force before usual retirement ages.^6^ Depending on the health outcome (eg, psychological or physical health), the study design and sample (eg, many earlier studies are based on non-representative occupational cohorts such as GAZEL and Whitehall), the country considered (USA or Europe), its timing, and the reasons given for leaving work (eg, voluntary, ill health, etc), retirement has been found to have beneficial, detrimental or no effects on health.^7–15^ The evidence largely suggests that retirement is associated with an improvement in psychological health and well-being,^7–9^ ^15^ but results are less consistent for physical health.^9–15^

However, working post-SPA is a relatively recent trend and so, until now, few studies have focused on the health consequences of continuing work *past* statutory retirement age while accounting for both health selection and labour market attachment prior to SPA.^6^ For example, a report using the UK British Household Panel Survey suggests that those working beyond SPA report better self-rated health.^16^ However, this study failed to control for health selection into work in later life, a critical limitation given research showing that healthier people are more likely to remain in paid employment, particularly at older ages.^17^ Calvo *et al*,^15^ in one of the few studies to explicitly consider the health effects of continuing work past expected retirement age, as well as accounting for health selection (using an instrumental variable approach), found that older Americans who continued working past 62 (the eligibility age for claiming early Social Security retirement benefits) reported no health benefits, relative to those who retired at this age.

Given recent trends towards working longer, further research is needed on the health consequences of continuing to work *past statutory retirement age*. Moreover, it is critical to account for earlier health status, but life-course research suggests that lifetime labour market attachment is also likely to affect the relationship between paid work and later life health.^18–23^ For example, McMunn *et al*^21^ show that British women who combine family roles with strong labour market attachment are healthier in their mid-50s than those who spend long periods of time out of the labour market looking after the home and family, independent of social position or health earlier in adulthood. Prior employment experiences may also shape decisions about later life work; for example, some may extend their working lives to compensate for earlier interruptions and periods out of the labour market.^24^

Against this background, our study draws on a life-course perspective to address two specific research questions. First, we investigate whether or not paid work beyond SPA has a beneficial effect on the health of older adults in England once both earlier health status and lifetime labour market participation are taken into account. Second, we study whether this association differs by hours worked, type of job and social class. We investigate three measures of health: depression, sleep disturbance and somatic health. These indicators of health are all associated with increased mortality and worsened quality of life, even after related covariates are controlled for.^25–28^

## Methods

### Study population

We used data from the second (2004/2005), third (2006/2007) and fourth waves (2008/2009) of the English Longitudinal Study of Ageing (ELSA), a multidisciplinary longitudinal survey of individuals aged 50 and over living in private households in England (http://www.elsa-project.ac.uk/). Wave 1 data were excluded because nurse-measured health indicators (eg, grip strength) were collected in alternate waves beginning in wave 2.

Our initial sample included respondents who had reached the current SPA (65 for men, 60 for women)^29^ by wave 3 and who participated in wave 2. This sample was then further restricted to men aged 65–74 and women aged 60–69 at wave 3. This is because few men and women work beyond 74 and 69, respectively. We also included respondents who participated in wave 4 (ie, excluding those who died n=36, 1.5%) for an initial sample size of about 2300 respondents. Of these, however, only 2010 (about 86%) took part in the life history interview administered at wave 3, when retrospective histories with information about employment experiences in adulthood and about health in childhood and adulthood were collected. The sample was also further reduced due to loss to follow-up and to missing nurse visit information at wave 4. A total of N=2039 respondents had information collected in waves 2, 3 and 4 (but not necessarily at the life history interview); and N=1811 respondents were present also in the nurse visit at follow-up (although they might have not taken part in the life history interview). In total, N=1608 respondents (ie, about 69% of the initial sample) were present in all waves, including nurse visits and the life history interview.

### Measures

#### Outcomes (wave 4)

Our health outcomes are depression, sleep disturbance and somatic health. Symptoms of depression were measured by an abbreviated eight-item version of the validated Centre for Epidemiologic Studies Depression Scale.^30^ Respondents who reported three or more depressive symptoms in the week prior to the interview were classified as being depressed.^31^ Sleep disturbance was assessed with three questions about whether, in the past month, respondents had difficulties falling or staying asleep, and whether they felt tired on waking up. Responses were given a numerical score (ranging from 1=‘not during the last month’ to 4=‘three or more times a week’). As in previous studies, respondents with a score in the lowest sex-specific quartile were categorised as having disturbed sleep.^32^ Finally, as a measure of somatic health, we derived a latent health index using a similar procedure to that proposed by Ploubidis and Grundy.^33^ This index combines both self-reported health (self-rated health (SRH); presence of one or more limitations with activities of daily living (ADL); severe long-standing illness; self-reports of doctor-diagnosed heart disease and stroke; mobility limitations) and nurse-measured information (grip strength). All indicators were recoded such that high values represent good health. Our latent health index assumes that these indicators reflect an underlying common dimension of health, which we refer to as somatic health following Ploubidis and Grundy,^33^ although we recognise that psychological health is also likely to influence an individual's self-assessment of his/her health. This derived latent health index is less subject to measurement error than separate health indicators, and therefore has greater repeatability and reliability.^33^ ^34^

#### Paid work beyond SPA (wave 3)

Our key independent variable was a binary indicator which distinguishes respondents by whether they are in paid work or not beyond SPA. Those who reported paid work or self-employment in the month prior to interview at wave 3 were classified as ‘in paid work’. Those ‘not in paid work’ mostly reported being retired (almost 90% overall). The remaining 10% could not be considered in more detail given the small number of respondents who classified themselves as sick, homemakers or unemployed.

We also considered alternative measures of paid work which account for work characteristics including working hours, type of work and social class, using them in essence as effect modifiers. In particular, we classified respondents in paid work by working hours/week (20+ hours vs <20 hours); by level of physical effort involved in their jobs (sedentary occupations vs jobs involving physical exertion); and by social class (distinguishing between higher managerial, administrative and professional occupations; intermediate occupations; and routine and manual occupations following the National Statistics Socio-Economic Classification (NS-SEC)).

#### Confounders

##### Life course (ELSA life history data)

Individual employment histories—the series of labour market statuses between the ages of 16 and state pension eligibility (64 for men and 59 for women)—were modelled using optimal matching analysis.^35^ In particular, we used an ‘ideal type’ derivation which compares all observed sequences of work events against a set of ideal type trajectories,^36^ as described fully in Corna *et al*.^37^ We considered five ideal employment histories for men (employed full-time throughout; not employed throughout; full-time up to 59; early exit at 49; and start of paid work at 23 and exit at 60) and seven for women (employed full-time throughout; employed mostly part time throughout; not employed throughout; early exit at 48; with a short career break between 26 and 30 followed by part time employment; with a long career break between 26 and 41 followed by part time employment; and with a medium career break between 26 and 34 followed by full-time employment). It is important to note that individuals in each of these categories are mostly/always employed or non-employed around the ages indicated because cases are matched to their closest model sequence. Moreover, as controls for life-course health selection, the life histories in wave 3 provided information on health in childhood (SRH, dichotomised as good, very good or excellent health vs fair or poor) and adulthood (number of periods of ill health or disability lasting more than a year, recoded into two or more periods of ill health vs one or none).

##### Baseline (wave 2)

Age, educational level, marital status, wealth, housing tenure, caring responsibilities and health behaviours at baseline (smoking and vigorous physical activity) were included in our analyses as important confounders of the association between paid work post-SPA and health in later life.^3–5^ ^7–15^ Educational level was recoded into three categories (low, middle, high) using the International Standard Classification of Education (http://www.uis.unesco.org/), where low education refers to below secondary education and a high educational level is defined as having a university education or above. Marital status categories initially distinguished married or cohabiting participants from the never-married, widowed, and separated or divorced. However, given the small number of never-married and widowed respondents in paid work, in our final models we only used a dichotomous variable (married or cohabiting vs unmarried). Wealth is the total net non-housing wealth indicator created by the Institute for Fiscal Studies;^38^ we categorised respondents by whether or not they were in the highest wealth quintile. Housing tenure was recoded into a three-category variable distinguishing outright owners, owners with a mortgage and non-owners. Caring responsibilities were defined as caring for someone in the week prior to the interview; we distinguished between respondents who cared for someone for at least 15 hours/week from those who did so less often, and those who did not provide care. In our final model for men, however, we only considered a dichotomous variable for care and did not consider the number of hours of care provided. Finally, in addition to depression and the latent somatic health measures at baseline (see above for derivation), we also controlled for smoking (whether or not a current smoker) and vigorous physical activity (once a week or more compared with less often). It was not possible to control for sleep disturbance at baseline as this was not collected at wave 2.

### Statistical analyses

We assessed the longitudinal relationship between paid work at wave 3 and health at wave 4, controlling for demographic characteristics, socioeconomic factors and health behaviours at wave 2 (baseline), and employment histories as potential confounders. Importantly, we also adjust for health throughout the life course to account for health selection into paid work beyond SPA. In the light of significant gender differences in labour market histories, health status and SPA in England, we carried out analyses separately for men and women. We also repeated analyses by type of work, working hours and social class as described above in the Paid work beyond SPA (wave 3) section. We used logistic regression when depression and sleep disturbance were considered, and linear regression for the continuous somatic health variable. Models were initially estimated using complete case analyses (not shown). However, about up to 21% of the sample had at least one missing value (particularly with respect to the life history information). Using the multiple imputation (MI) approach which assumes that data are missing at random rather than completely at random,^39^ ^40^ we ran imputations separately by gender using chained equations (20 cycles; repeated independently 50 times).The results of these analyses were then combined using Rubin's rules.^39^ Since the results for the complete case and the imputed data sets were broadly similar, in this paper we present models for the imputed data sets restricted to respondents who had complete information for the health outcomes considered. Latent summaries were estimated using Mplus 7.3; all analyses were performed in STATA V.13.

## Results

### Measurement model for the latent measure of somatic health

In order to summarise measures of somatic health, we considered a unidimensional model, where a single latent factor accounts for variation in self-reported and observer-measured health indicators. As shown in online supplementary table A, all health indicators significantly loaded on the latent factor, with good self-rated health, and the absence of ADL, mobility limitations and severe long-standing illness having the strongest standardised factor loadings (with values around 0.80). Goodness of fit criteria indicate that the model fits the data well. The latent variable obtained by combining the health indicators offers a continuous health measure, where positive high scores indicate good somatic health. Its estimated distribution is essentially Gaussian (mean=−0.04; SD=0.74), although slightly skewed left (skewness=−0.34).

10.1136/jech-2016-208086.supp1 supplementary table

### Descriptive statistics

The characteristics of the male and female respondents who were successfully interviewed at wave 2, wave 3 and in the life history; and who were aged 65–74 and 60–69, respectively, also at wave 3 (N=2010) are shown in table 1. About 25% of older women and 15% of men reported working beyond SPA. This is comparable to data from the Labour Force Survey which shows that 22% of women aged 60–69 and 14% of men aged 65–74 were in paid employment in the UK in 2006. Overall, men and women in good health were more likely to be in paid work post-SPA at wave 3, as were those in better health throughout their lives (ie, had fewer than two spells of ill health in adulthood, and good health in childhood), and those with postsecondary education. Marital status, home tenure and caring responsibilities at baseline were associated with work post-SPA only among women: female respondents divorced or separated, those with an unpaid mortgage, and those who did not provide care were more likely to be in paid work post-SPA at wave 3.

**Table 1:** Per cent (and N) distribution of baseline and life history health, demographic, and socioeconomic characteristics, by gender and paid work post state pension age

	Men			Women		
	Not in paid work at wave 3	In paid work at wave 3	p Value	Not in paid work at wave 3	In paid work at wave 3	p Value
Baseline (wave 2) characteristics						
Depression	15.1 (111)	10.3 (13)	NS	25.1 (216)	17.0 (49)	***
Sleep disturbance*	28.7 (187)	20.0 (24)	**	32.0 (252)	26.9 (71)	NS
Somatic health (mean)†	−0.026	0.345	***	−0.180	0.182	***
Not smoking	84.6 (620)	90.5 (114)	NS	84.4 (724)	84.7 (243)	NS
Vigorous physical activity 1+/week	22.7 (197)	21.1 (30)	NS	16.6 (167)	24.7 (83)	***
Age (mean)	69.7	68.5	***	64.7	62.7	***
Never married	5.3 (39)	1.6 (2)	NS	3.4 (29)	3.1 (9)	***
Married/cohabiting	77.9 (572)	84.9 (107)		69.3 (597)	68.5 (198)	
Divorced/separated	8.6 (63)	10.3 (13)		12.4 (107)	19.4 (56)	
Widowed	8.2 (60)	3.2 (4)		14.9 (128)	9.0 (26)	
No education	33.7 (246)	23.8 (30)	**	38.6 (331)	25.0 (72)	***
Some education	39.0 (270)	35.7 (45)		40.9 (351)	41.7 (120)	
High education	29.3 (214)	40.5 (51)		20.5 (176)	33.3 (96)	
Highest wealth quintile	18.9 (137)	27.9 (34)	**	19.6 (167)	23.9 (67)	NS
Own outright	75.4 (553)	71.4 (90)	NS	72.2 (621)	59.9 (173)	***
Mortgage	10.1 (74)	15.1 (19)		14.1 (121)	32.5 (94)	
Rent	14.5 (106)	13.5 (17)		13.7 (118)	7.6 (22)	
No care provided	90.7 (666)	94.4 (119)	NS	83.9 (722)	87.9 (254)	**
Cared <15 hours/week	5.3 (39)	4.0 (5)		7.2 (62)	8.0 (23)	
Cared 15+ hours/week	4.0 (29)	1.6 (2)		8.9 (77)	4.1 (12)	
Life course						
Ever left employer because of ill health	22.2 (163)	8.7 (11)	***	25.6 (220)	9.7 (28)	***
2 or more periods of ill health in adulthood	15.7 (115)	6.4 (8)	***	20.2 (174)	6.9 (20)	***
SRH as good, very good or excellent in childhood	88.3 (648)	92.0 (115)	NS	83.4 (717)	90.0 (260)	***
Total respondents (N)	85.4% (734)	14.6% (126)		74.9% (861)	25.1% (289)	

Source: ELSA 2004/2005, 2006/2007, 2008/2009, ELSA life history, nurse visit at wave 2. Own calculations; unweighted data.

p Values refer to the relevant statistical tests (ie, Student's t-test, ANOVA or χ^2^ tests); **, ***: significant at the 0.05 and 0.0l levels, respectively.

*Sleep disturbance was only collected at wave 4; therefore the associations displayed in this table are based on those respondents who provided answers about their quality of sleep at follow-up.

†Somatic health was calculated only for respondents who had a nurse visit at wave 2.

ANOVA, analysis of variance; ELSA, English Longitudinal Study of Ageing; NS, not significant.

### Current and lifetime paid work characteristics

**Figure 1:**
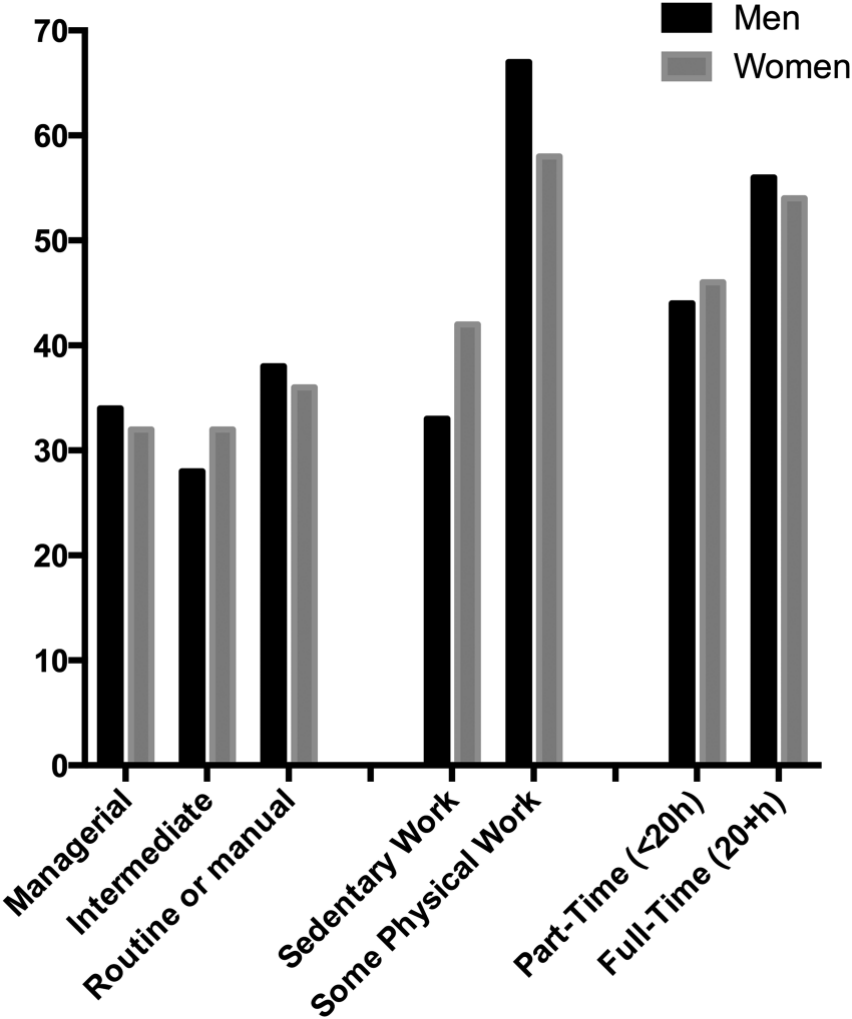
Percent distribution of work characteristics among respondents in paid work, by gender. Source: English Longitudinal Study of Ageing (ELSA), 2006/2007. Analyses restricted to male respondents aged 65–74 (N=126) and female respondents aged 60–69 (N=289) who were in paid work.

Figure 1 shows the employment characteristics of respondents who worked beyond SPA. About one-third were in managerial positions, almost half (45%) worked <20 hours/week, and one-third of male and 41% of female workers had a sedentary job. No significant gender differences were found in these characteristics.

Table 2 presents the classification of respondents’ employment histories for the sample and by whether they engaged in paid work post-SPA. Results suggest that men and women who worked throughout their lives up to SPA (full-time or part time, with or without career breaks) were more likely to be in paid work after SPA. However, given the small number of cases in some categories, we combined them in order to create categories that included respondents with conceptually similar employment histories. In our multivariate model, we considered only three categories for both men (continuous work up to SPA, continuous work up to about age 60, and weak labour market attachment) and women (continuous work either full-time or part time up to SPA, paid work up to SPA with family care interruptions of any length, and weak labour market attachment including leaving the labour market at about age 48).

**Table 2:** Per cent (and N) distribution of life-course labour market histories, by paid work post-SPA

	Not in paid work at W3	In paid work at W3	All sample
*Men*			
Continuous work up to SPA			
Mostly FT throughout to SPA	40.4 (296)	75.6 (93)	45.4 (389)
Weak labour market attachment			
Mostly non-employed throughout	4.9 (36)	0.8 (1)	4.3 (37)
FT very early exit (at about age 49)	12.7 (93)	4.1 (5)	11.5 (98)
Continuous work up to about age 60			
FT early exit (at about age 60)	33.6 (246)	5.7 (7)	29.6 (253)
Late start at about age 23, early exit (at about age 60)	8.5 (62)	13.8 (17)	9.2 (79)
*Women*			
Continuous work FT or PT up to SPA			
Mostly PT throughout to SPA	5.2 (45)	7.3 (21)	5.7 (66)
Mostly FT throughout to SPA	25.3 (217)	30.6 (88)	26.6 (305)
Weak labour market attachment			
Mostly non-employed throughout/family carer	21.4 (184)	4.2 (12)	17.1 (196)
Early exit (at about age 48)	9.7 (83)	0.7 (2)	7.4 (85)
Paid work up to SPA with family care interruptions			
Long break (about ages 26–41) to PT up to SPA	10.4 (89)	16.0 (46)	11.8 (135)
Short break (about ages 26–30) to PT up to SPA	11.4 (98)	18.8 (54)	13.3 (152)
Medium break (about ages 26–34) to FT up to SPA	16.6 (143)	22.6 (65)	18.1 (208)

Source: ELSA life history, 2006/2007.

ELSA, English Longitudinal Study of Ageing; FT, full-time; PT, part time; SPA, state pension age; W3, wave 3.

Per cent distribution of work characteristics among respondents in paid work, by gender (figure 1).

### Associations between work beyond SPA and health at follow-up

Table 3 shows the longitudinal associations between paid work beyond SPA and health for men and women, respectively. For each health outcome, we present unadjusted and fully adjusted results. The unadjusted estimates show that men and women in paid work were more likely to be in better health at follow-up than those not in paid work. For instance, respondents in paid work beyond SPA were between 0.44 (men) and 0.57 (women) times less likely to be depressed, and between 0.64 (men) and 0.73 (women) times less likely to report sleep disturbance. They were also significantly more likely to report better somatic health (β=0.323 for men, and β=0.292 for women). However, for all three health indicators considered, the beneficial effect of paid work was not observed once the other covariates were included.

**Table 3:** Unadjusted and fully adjusted ORs and β coefficients (with 95% CIs) for the relationship between paid work beyond SPA and health among men and women

	Depression		Sleep disturbance		Somatic health	
	Unadjusted model	Fully adjusted model*	Unadjusted model	Fully adjusted model*	Unadjusted model	Fully adjusted model*
*Men*						
In paid work	0.44 (0.22 to 0.90)	1.21 (0.53 to 2.75)	0.64 (0.41 to 1.00)	0.99 (0.60 to 1.67)	0.323 (0.207 to 0.439)	0.059 (−0.033 to 0.152)
Weak labour attachment†	3.64 (2.06 to 6.42)	1.76 (0.86 to 3.58)	1.62 (1.02 to 2.59)	0.98 (0.56 to 1.72)	−0.194 (−0.315 to −0.073)	−0.044 (−0.116 to 0.029)
Continuous work up to 59†	1.57 (0.94 to 2.59)	1.16 (0.64 to 2.10)	1.28 (0.90 to 1.81)	1.10 (0.74 to 1.65)	−0.121 (−0.212 to −0.031)	0.078 (−0.021 to 0.177)
Depression‡	12.7 (8.09 to 19.8)	7.57 (4.49 to 12.7)	3.68 (2.48 to 5.47)	2.28 (1.46 to 3.57)	−0.409 (−0.525 to −0.293)	−0.093 (−0.191 to 0.004)
Somatic health	0.27 (0.18 to 0.39)	0.56 (0.35 to 0.88)	0.33 (0.24 to 0.44)	0.45 (0.32 to 0.64)	0.692 (0.639 to 0.745)	0.704 (0.640 to 0.766)
Smoker§	2.59 (1.61 to 4.15)	1.33 (0.70 to 2.43)	1.87 (1.25 to 2.80)	1.28 (0.76 to 1.92)	−0.149 (−0.267 to −0.031)	−0.011 (−0.102 to 0.080)
No vigorous physical activity¶	2.61 (1.43 to 4.79)	1.77 (0.85 to 3.66)	2.24 (1.49 to 3.35)	1.50 (0.96 to 2.33)	−0.318 (−0.414 to −0.222)	−0.089 (−0.165 to −0.013)
Age	0.98 (0.91 to 1.04)	0.97 (0.89 to 1.06)	0.98 (0.93 to 1.03)	0.96 (0.90 to 1.02)	−0.024 (−0.038 to −0.009)	−0.013 (−0.024 to −0.001)
Not married**	2.93 (1.93 to 4.45)	1.99 (1.18 to 3.39)	1.28 (0.89 to 1.82)	0.91 (0.60 to 1.40)	−0.103 (−0.205 to −0.021)	−0.023 (−0.105 to 0.057)
No education††	2.81 (1.62 to 4.86)	1.09 (0.53 to 2.23)	2.09 (1.41 to 3.09)	1.29 (0.81 to 2.04)	−0.361 (−0.463 to −0.258)	−0.136 (−0.224 to −0.049)
Some education††	1.85 (1.06 to 3.25)	1.54 (0.80 to 2.99)	1.39 (0.94 to 2.05)	1.18 (0.77 to 1.80)	−0.144 (−0.243 to −0.045)	−0.037 (−0.116 to 0.041)
Not in the highest wealth quintile‡‡	3.26 (1.61 to 6.58)	2.11 (0.94 to 4.74)	1.71 (1.14 to 2.58)	1.07 (0.67 to 1.70)	−0.302 (−0.404 to −0.198)	−0.056 (−0.138 to 0.027)
Mortgage§§	0.98 (0.48 to 1.97)	0.78 (0.34 to 1.79)	0.89 (0.53 to 1.48)	0.75 (0.43 to 1.30)	−0.021 (−0.155 to 0.113)	0.007 (−0.096 to 0.109)
Rent§§	3.43 (2.15 to 5.46)	1.06 (0.57 to 2.00)	2.07 (1.37 to 3.11)	1.10 (0.67 to 1.79)	−0.238 (−0.358 to −0.118)	0.068 (−0.030 to 0.164)
Cared for sick/disabled adult¶¶	1.62 (89 to 2.96)	2.57 (1.24 to 5.31)	0.99 (0.59 to 1.66)	1.13 (0.64 to 1.92)	0.165 (0.020 to 0.311)	0.061 (−0.047 to 0.170)
2+ periods of ill health***	3.50 (2.15 to 5.69)	1.94 (1.06 to 3.57)	2.45 (1.61 to 3.72)	1.48 (0.98 to 2.36)	−0.561 (−0.674 to −0.447)	−0.216 (−0.310 to −0.121)
SRH in childhood=fair/poor†††	2.33 (1.34 to 4.06)	1.94 (1.03 to 3.68)	1.26 (0.78 to 2.05)	1.00 (0.60 to 1.67)	−0.256 (−0.387 to −0.125)	−0.091 (−0.189 to 0.004)
Total respondents (N)	867		867		766	
*Women*						
In paid work	0.57 (0.40 to 0.80)	1.03 (0.67 to 1.58)	0.73 (0.54 to 0.97)	1.05 (0.72 to 1.50)	0.292 (0.209 to 0.374)	0.004 (−0.062 to 0.071)
Weak labour attachment†	1.31 (0.91 to 1.89)	1.09 (0.67 to 1.78)	0.99 (0.70 to 1.41)	0.75 (0.50 to 1.15)	−0.103 (−0.200 to −0.006)	0.004 (−0.004 to 0.011)
Paid work up to SPA with family care interruptions†	0.68 (0.48 to 0.96)	0.91 (0.58 to 1.41)	0.83 (0.61 to 1.21)	1.05 (0.72 to 1.53)	0.125 (0.040 to 0.210)	0.012 (−0.053 to 0.077)
Depression‡	6.57 (4.85 to 8.91)	4.35 (3.10 to 6.09)	3.28 (2.47 to 4.37)	2.10 (1.52 to 2.90)	−0.479 (−0.561 to −0.396)	−0.101 (−0.167 to −0.034)
Somatic health	0.29 (0.22 to 0.37)	0.46 (0.33 to 0.63)	0.29 (0.23 to 0.37)	0.33 (0.24 to 0.44)	0.728 (0.685 to 0.771)	0.689 (0.636 to 0.741)
Smoker§	1.69 (1.19 to 2.40)	1.27 (0.83 to 1.92)	0.96 (0.67 to 1.35)	0.72 (0.52 to 1.21)	−0.196 (−0.297 to −0.096)	−0.036 (−0.110 to 0.037)
No vigorous physical activity¶	1.92 (1.29 to 2.83)	1.13 (0.73 to 1.77)	1.18 (0.85 to 1.62)	0.68 (0.49 to 0.98)	−0.320 (−0.411 to −0.229)	−0.067 (−0.135 to −0.001)
Age	1.03 (0.98 to 1.08)	1.03 (0.97 to 1.09)	0.98 (0.94 to 1.02)	0.97 (0.92 to 1.02)	−0.013 (−0.025 to −0.001)	−0.006 (−0.016 to 0.003)
Not married**	1.54 (1.16 to 2.05)	1.01 (0.72 to 1.43)	1.19 (0.86 to 1.46)	0.88 (0.64 to 1.20)	−0.137 (−0.215 to −0.058)	−0.029 (−0.089 to 0.031)
No education††	1.47 (1.02 to 2.12)	0.82 (0.53 to 1.29)	1.55 (1.10 to 2.19)	1.25 (0.83 to 1.87)	−0.217 (−0.314 to −0.121)	−0.028 (−0.102 to 0.045)
Some education††	1.23 (0.85 to 1.78)	0.99 (0.65 to 1.50)	1.64 (1.17 to 2.29)	1.51 (1.05 to 2.19)	−0.111 (−0.205 to −0.017)	−0.020 (−0.087 to 0.049)
Not in the highest wealth quintile‡‡	1.99 (1.35 to 2.95)	1.16 (0.73 to 1.82)	1.72 (1.22 to 2.40)	1.17 (0.80 to 1.71)	−0.300 (−0.390 to −0.210)	−0.037 (−0.105 to 0.032)
Mortgage§§	0.97 (0.67 to 1.40)	0.87 (0.63 to 1.50)	1.10 (0.80 to 1.52)	0.99 (0.69 to 1.42)	−0.011 (−0.105 to 0.083)	−0.003 (−0.073 to 0.067)
Rent§§	2.47 (1.69 to 3.58)	1.79 (1.14 to 2.82)	1.72 (1.20 to 2.48)	1.36 (0.88 to 2.09)	−0.299 (−0.412 to −0.187)	−0.036 (−0.121 to 0.049)
Cared <15 hours/week¶¶	1.25 (0.76 to 2.03)	1.54 (0.88 to 2.66)	0.90 (0.56 to 1.44)	0.96 (0.57 to 1.60)	0.003 (−0.137 to 0.144)	−0.060 (−0.159 to 0.039)
Cared 15+ hours/week¶¶	0.99 (0.59 to 1.63)	1.17 (0.66 to 2.09)	0.69 (0.42 to 1.12)	0.67 (0.38 to 1.16)	−0.085 (−0.222 to 0.053)	−0.138 (−0.234 to −0.041)
2+ periods of ill health***	2.28 (1.61 to 3.22)	1.17 (0.76 to 1.78)	2.17 (1.56 to 3.02)	1.11 (0.73 to 1.68)	−0.642 (−0.732 to −0.552)	−0.253 (−0.328 to −0.179)
SRH in childhood=fair/poor†††	1.78 (1.23 to 2.58)	1.22 (0.81 to 1.86)	1.67 (1.18 to 2.38)	1.19 (0.81 to 1.75)	−0.278 (−0.380 to −0.176)	−0.005 (−0.079 to 0.069)
Total respondents (N)	1172		1172		1045	

Source: ELSA 2004/2005, 2006/2007, 2008/2009, ELSA life history, nurse visits at wave 2 and wave 4.

*The fully adjusted model controls for wave 2 demographic and socioeconomic characteristics, labour market history and life-course health.

Reference categories †continuous work up to 59 years of age; ‡no depression at W2; §not a smoker at W2; ¶vigorous physical activity at W2; **married or cohabiting; ††high education; ‡‡in the highest wealth quintile; §§own outright; ¶¶no care provided; ***1 or no periods of ill health in adulthood; †††SRH in childhood=good, very good, excellent. Own calculations.

ELSA, English Longitudinal Study of Ageing; SPA, state pension age; W2, wave 2; SRH, self-rated health.

In the fully adjusted models, baseline and life-course health covariates remained significantly associated with the three outcomes. Having had two or more periods of illness in adulthood increased the odds of reporting depression and sleep disturbance among men by a factor of 1.94 and 1.48, respectively; male (β=−0.216) and female (β=−0.253) respondents also were in significantly poorer somatic health. Finally, unadjusted results showed negative associations between weak labour market attachment and good health for men (all health outcomes) and women (for somatic health only).

Table 4 shows results obtained when alternative characteristics of paid work were considered. Fully adjusted results suggest that paid work beyond SPA does not have differential effects on health depending on characteristics of the job such as physical demand, hours worked or social class.

**Table 4:** Unadjusted and fully adjusted ORs and β coefficients (with 95% CIs) for the relationship between three different characteristics of paid work beyond state pension age and health among men and women

	Depression		Sleep disturbance		Somatic health	
	Unadjusted model	Fully adjusted model*	Unadjusted model	Fully adjusted model*	Unadjusted model	Fully adjusted model*
*Men*						
In work, sedentary	0.46 (0.14 to 1.53)	1.88 (0.52 to 6.81)	0.49 (0.21 to 1.13)	0.96 (0.39 to 2.37)	0.367 (0.176;0.557)	0.008 (−0.141 to 0.157)
In work, physical	0.44 (0.19 to 1.03)	0.98 (0.38 to 2.49)	0.71 (0.42 to 1.20)	0.98 (0.54 to 1.77)	0.302 (0.162;0.440)	0.051 (−0.061 to 0.162)
Not in paid work	*REF*	*REF*	*REF*	*REF*	*REF*	*REF*
In work, part time	0.43 (0.15 to 1.22)	1.11 (0.37 to 3.29)	0.62 (0.32 to 1.18)	0.91 (0.45 to 1.85)	0.307 (0.137;0.476)	0.061 (−0.071 to 0.192)
In work, full-time	0.47 (0.18 to 1.18)	1.25 (0.45 to 3.48)	0.67 (0.37 to 1.21)	1.05 (0.54 to 2.03)	0.332 (0.182;0.482)	0.010 (−0.110 to 0.129)
Not in paid work	*REF*	*REF*	*REF*	*REF*	*REF*	*REF*
In work, managerial	0.62 (0.22 to 1.77)	1.46 (0.42 to 6.32)	0.85 (0.41 to 1.71)	1.60 (0.75 to 3.59)	0.311 (0.122;0.499)	−0.051 (−0.200;0.097)
In work, intermediate	0.43 (0.20 to 1.94)	1.09 (0.36 to 3.42)	0.37 (0.14 to 0.97)	0.59 (0.22 to 1.59)	0.259 (0.048;0.470)	0.048 (−0.112;0.210)
In work, routine/manual	0.67 (0.26 to 1.73)	1.12 (0.37 to 3.31)	0.69 (0.34 to 1.38)	0.87 (0.40 to 1.89)	0.380 (0.201;0.559)	0.105 (−0.037;0.247)
Not in paid work	*REF*	*REF*	*REF*	*REF*	*REF*	*REF*
Total respondents (N)	867		867		766	
*Women*						
In work, sedentary	0.51 (0.30 to 0.86)	0.98 (0.54 to 1.78)	0.92 (0.61 to 0.19)	1.40 (0.86 to 2.26)	0.307 (0.188 to 0.426)	−0.018 (−0.109;0.072)
In work, physical	0.62 (0.40 to 0.95)	1.03 (0.63 to 1.70)	0.59 (0.40 to 0.87)	0.80 (0.52 to 1.24)	0.281 (0.178 to 0.383)	0.013 (−0.065 to 0.092)
Not in paid work	*REF*	*REF*	*REF*	*REF*	*REF*	*REF*
In work, part time	0.53 (0.31 to 0.87)	0.86 (0.49 to 1.51)	0.77 (0.51 to 1.18)	1.02 (0.64 to 1.62)	0.261 (0.147;0.376)	0.013 (−0.073 to 0.115)
In work, full-time	0.62 (0.40 to 0.96)	1.21 (0.72 to 2.05)	0.68 (0.47 to 1.00)	1.01 (0.64 to 1.60)	0.328 (0.221 to 0.434)	0.003 (−0.082 to 0.087)
Not in paid work	*REF*	*REF*	*REF*	*REF*	*REF*	*REF*
In work, managerial	0.37 (0.19 to 0.73)	0.83 (0.40 to 1.74)	0.83 (0.51 to 13.4)	1.55 (0.86 to 2.80)	0.401 (0.27;0.534)	0.045 (−0.061 to 0.151)
In work, intermediate	0.75 (0.45 to 1.25)	1.35 (0.73 to 2.47)	0.82 (0.51 to 1.30)	1.09 (0.65 to 1.87)	0.257 (0.123 to 0.390)	−0.026 (−0.124 to 0.074)
In work, routine/manual	0.58 (0.33 to 1.00)	0.86 (0.45 to 1.61)	0.57 (0.35 to 0.94)	0.70 (0.41 to 1.18)	0.226 (0.101 to 0.352)	−0.010 (−0.107 to 0.085)
Not in paid work	*REF*	*REF*	*REF*	*REF*	*REF*	*REF*
Total respondents (N)	1172		1172		1045	

Source: English Longitudinal Study of Ageing (ELSA) 2004/2005, 2006/2007, 2008/2009, ELSA life history, nurse visits at wave 2 and wave 4.

*The fully adjusted model controls for wave 2 demographic and socioeconomic characteristics (age, education, wealth, marital status, housing tenure, caring responsibilities, depression, somatic activity and health behaviours); labour market history; and health at childhood and adulthood. Own calculations.

## Conclusion and discussion

Many Western societies are raising SPA. However, the health effects of extending employment are not fully understood. Using a life-course approach, this study investigated whether employment beyond SPA is beneficial for health once both prior health status and work histories are taken into account. ELSA's rich data and large representative sample allowed us to adjust for some key life-course health characteristics as well as labour market histories that may confound the association between work beyond SPA and subsequent somatic and psychological health. Similar to Calvo *et al*'s^15^ findings, our results also suggest that being in paid work beyond SPA is not associated with better (or detrimental) health. This is most likely because only a select group of healthy older adults works beyond SPA.

Our contribution should be considered in the light of several limitations. First, although a number of different elements of paid work were considered, we were not able to fully capture other key dimensions of work beyond SPA. For instance, we do not know the reasons why respondents worked beyond SPA as this information was not collected until wave 4. We were also not able to explore effort and reward imbalances at work as these questions were only asked in the drop-off questionnaire (and this would have further reduced the analytical sample size). Moreover, in constructing labour market histories, we clustered individuals whose trajectories are similar but not identical, and thus we cannot say whether this ‘muddied’ associations with subsequent health or with the other independent variables. Second, it was not possible to consider lifetime social class as information on usual social class is not available in ELSA. Third, among life-course health controls, we were limited by data availability: we were not able to consider the presence or the timing of the onset of specific health conditions, but we should also acknowledge that the measures of health in childhood and adulthood used are rather broad proxies for pre-SPA health. Also, while in our analysis baseline characteristics were considered confounders, some of these same variables might act as mediators/effect modifiers if measured after SPA (for instance, caring might mediate/moderate the working to health relationship). Finally, a 2-year period may have been too short to observe a relationship between paid work beyond SPA and subsequent health.

In summary, our analysis suggests that extending working lives may not have beneficial effects on health. The decision and the ability to continue working beyond SPA seems to be strongly affected by prior health. Respondents with two or more periods of ill health were most likely to report that they experienced such problems in their 50s and 60s, with about one-third reporting that periods of ill health limited their opportunities for paid work. However, these findings do not rule out the possibility that changes to SPA may worsen population health, if everyone is required to work longer, including people in poor health.

Although more research is needed to investigate when ill health matters most for work exits, and whether our results will also hold for future generations, our analyses suggest that the ability to work longer hinges on both current and lifetime health. Optimising and promoting health throughout the life course seems key to policies aimed at extending working lives.

**What is already known on this subject**

- Participation in paid work is associated with good health in adulthood. However, little work has examined the health implications of labour market participation among those who continue to work post state pension age (SPA).
- Evidence on the impact of retirement on health largely suggests beneficial effects for psychological well-being (results are less consistent for physical health), even when health selection is addressed. However, most studies focus on early retirement with few explicitly investigating how continuing work beyond SPA affects later life health.
- In addition, few studies have explicitly examined the relationship between working beyond SPA and later life health while also considering both earlier health status and lifetime labour market experience.

**What this study adds**

▸ Adopting a life-course approach, this research study investigates whether employment beyond SPA is beneficial for health, using nationally representative data on England and considering health selection effects into paid work and life-course labour market participation.

▸ Our results suggest no beneficial or detrimental effects of continuing to work past statutory retirement age when both earlier health status and work histories are taken into account. This is most likely because only a select group of healthy older adults work beyond statutory retirement age.
