# Supplementary material for: Is being in paid work beyond state pension age beneficial for health? Evidence from England using a life-course approach
Source: J Epidemiol Community Health. 2017 Apr 10;71(5):431–8. doi: 10.1136/jech-2016-208086 (PMC5484027; doi:10.1136/jech-2016-208086)
Supplement: supplementary table [file jech-2016-208086supp001.pdf]

Supplementary Table A. Standardised factor loadings of the health indicators on the somatic health measurement model and descriptive criteria of model fit, at both baseline and follow-up.

Health indicators	Somatic health at baseline	Somatic health at follow-up
Grip strength	0.44***	0.37***
Self-rated health	0.77***	0.80***
ADL limitation	0.81***	0.80***
Severe long standing illness	0.81***	0.79***
Stroke	0.49***	0.43***
Heart condition	0.42***	0.35***
Mobility limitations	0.88***	0.85***
**Criteria of Model Fit**		
Comparative Fit Index	0.978	0.970
Tucker Lewis Index	0.972	0.958
Root Mean Square Error of Approximation (RMSEA)	0.051	0.057

Notes: All indicators were recoded such that high values represent good health. Values of the Comparative Fit Index and of the Tucker Lewis Index greater than 0.95 indicate good fit; values of the Root Mean Square Error of Approximation less than 0.06 indicate good fit.

***: significant at p<0.001
